# Relationships between Perceived Stress, Depression and Alcohol Use Disorders in University Students during the COVID-19 Pandemic: A Socio-Economic Dimension

**DOI:** 10.3390/ijerph17238853

**Published:** 2020-11-28

**Authors:** Beata Gavurova, Viera Ivankova, Martin Rigelsky

**Affiliations:** 1 Center for Applied Economic Research, Faculty of Management and Economics, Tomas Bata University in Zlin, 5139, 760 00 Zlin, Czech Republic; 2 Faculty of Management, University of Presov in Presov, 16, 080 01 Presov, Slovakia; viera.ivankova@smail.unipo.sk (V.I.); martin.rigelsky@smail.unipo.sk (M.R.)

**Keywords:** alcohol dependence, depression, stress, behavioural disorders, mental health, gender and income differences, socio-economic effects, isolation, COVID-19 coronavirus pandemic, Slovak students

## Abstract

The objective of the study was to examine the effects of perceived stress on depression and subsequently to examine the effects of depression on alcohol use disorders. The data were obtained by an electronic questionnaire survey during the coronavirus disease 2019 (COVID-19) pandemic (*n* = 1523 Slovak university students). Descriptive, regression, and correlation analysis were used in the analytical processing, while the analyses included students’ scores in three diagnostic tools (Perceived Stress Scale (PSS), Patient Health Questionnaire for depression (PHQ 9), and Alcohol Use Disorders Identification Test (AUDIT)), as well as gender and income characteristics. The PSS identified an increased level of perceived stress in female students, while in contrast, the AUDIT showed an increased level of alcohol use disorders in male students. Differences in mental and behavioural disorders between the gender and income categories were significant in most of the analysed cases. In terms of gender-income characteristics, it was possible to confirm a significant positive effect of the PSS score on the PHQ 9 score, as well as a significant positive effect of the PHQ 9 score on the AUDIT score. As a result, efforts to reduce stress will be reflected in a reduction of depressive disorders as well as a reduction of excessive alcohol consumption among students.

## 1. Introduction

Mental health and unhealthy patterns of behaviour of university students have an undeniable place in professional and public discussions, as this population group forms a society that will be the driving force of the economy in the future. University students often face situations that can be risky in terms of psychiatric disorders and substance use [1]. The alarming prevalence of stress and depressive symptoms among university students contributes to the importance of research in this issue [2]. In this context, it can be noted that up to 53% of students may suffer from depression, which can result in suicidal thoughts with fatal consequences [3]. Thus, the vulnerability of university students to stress [4,5] and depression [6] is obvious. At the same time, risky behaviour is often attributed to this age group, and excessive alcohol consumption is no exception [7,8]. In addition, the evidence shows that the risk of problem alcohol consumption is higher among university students compared to non-students in the same age group [1,9]. All these facts point to the urgent need to address these difficulties for students.

Stress, depression, and alcohol abuse are serious disorders that affect individuals themselves, but also society as a whole, leading to the idea that this issue can also be considered from a socio-economic point of view. In this context, it has been confirmed that the mental illness of individuals is an economic burden, especially for their families, both in terms of treatment costs and in terms of reducing their productivity, which affects society [10]. According to Dewa and McDaid [11], people with mental disorders have fewer employment opportunities, and poor mental health affects their ability to do their job. As a result, mental disorders may affect the economy in terms of unemployment, reduced productivity, and disability [11]. In terms of costs, depression and other mental disorders are among the diseases with the highest economic burden [12], and expenditure on mental health care and treatment also contributes to this burden [13,14,15]. In the socio-economic context, alcohol use is also a serious problem in terms of health-related costs and lost productivity [16], while alcohol dependence plays a significant role [17]. The economic burden can be associated with alcohol dependence, as people with alcohol dependence cause excessive costs in the economy [18]. Alcohol use and alcohol use disorders can be considered a major risk factor for many serious diseases and injuries, avoidable premature mortality, disability-adjusted life-years, but also increased health care expenditure and lost productivity, which undoubtedly affects the economic prosperity of countries and the well-being of their populations [19,20,21,22]. Based on these findings, the mental health and risky behaviour of the population should not to be overlooked. At the same time, if these disorders occur at a young age, it may place an even more serious burden on society and economic life in the future.

These facts encourage many researchers to examine the issue of mental health and unhealthy patterns of behaviour in a population group of university students, and their significant findings are presented in the following section of this study. The importance of this issue is also captured in the presented study, the purpose of which was to examine the mental well-being and risky behaviour of Slovak university students in the socio-economic context of their perceived stress, depression, and alcohol consumption. This study focuses not only on mapping the current situation and examining the effects within the analysed mental and behavioural disorders, but especially on identifying possible practical implications and interventions that may improve the situation in the future.

## 2. Theoretical Background

The introduction of this study suggests that mental and behavioural disorders in university students are a serious problem that should be addressed throughout society. In addition, the COVID-19 pandemic has adversely contributed to this problem, as students experienced increased stress, anxiety, and depressive thoughts during this pandemic situation [23]. According to Son et al. [23], the main reasons were worries about their health and the health of their family and friends, difficulty concentrating, sleep disorders, reduced social interactions, and increased academic concerns.

On this basis, it seems very beneficial to examine the relations between perceived stress, depression, and alcohol use disorders in Slovak university students against the background of the COVID-19 pandemic.

### 2.1. Perceived Stress

As already mentioned, stress appears to be a serious problem for university students as a very vulnerable group of the population [4,5]. In addition, there is evidence that perceived stress is negatively related to students’ health-related quality of life [24] and their perceived health status [25]. Stressed students suffer from many other health problems, including physical exhaustion, sleep disorders, irascibility, negative thoughts, and feelings of nervousness [26]. The fact that unhealthy behavioural decisions, mental disorders, and burnout are attributed to perceived stress increases the severity of the problem [27,28,29,30]. For these reasons, the perceived stress of university students should be measured and addressed.

The Perceived Stress Scale (PSS) is a recognized diagnostic tool that is commonly used to measure stress levels, especially in research focused on the role of stress in the aetiology of diseases and behavioural disorders [31]. Orucu and Demir [32] recommended the use of this tool across different cultures and also in the student population. This tool consists of a 14-item scale, but its 10-item version is widely used in international studies examining students’ perceived stress [33,34,35,36,37]. A higher score generally indicates a higher perceived stress. In the Slovak Republic, the reliability and validity of this tool was verified by Ráczová et al. [38], who supported its use in a sample of the Slovak population. Last but not least, a 4-item version of PSS is also used, based on which Orosova et al. [39] revealed that 37.6% of Slovak students suffered from lower stress, approximately the same proportion (37.7%) suffered from moderate stress, and 24.7% of Slovak students experienced higher stress.

In this area of research, many international studies deal with the stress of students of medical disciplines, making them a special category. The 10-item PSS tool was also often used in these studies, and it can be stated that medical students reported moderate levels of stress [40]. In the Russian study [41], medical and dental students acquired a mean score of 16.97, with gender and socio-economic status playing a significant role in the differences in scores. Polish medical students obtained a mean PSS score of 22.78 [42]. In another international study of nursing students, Slovak, Polish, and Spanish students reported similar mean values for the 10-item PSS score, which indicated moderate stress (the Slovak Republic = 18.66, Poland = 18.60, Spain = 18.55) [43]. In this context, there is evidence that medical students have a higher risk of stress compared to non-medical students [44]. On the other hand, the evidence shows that the 10-item PSS score differs not only in fields of study, but also in various countries. Based on the mean PSS scores obtained in international studies, moderate to high stress was measured in students from the United States (US) [25,45], Italy [46], Korea [30], and the United Kingdom (UK) [47].

At the same time, it is necessary to emphasize the fact that the dominance of female students in the obtained higher level of stress measured by the 10-item PSS tool has been proven in many studies [30,44,46,48]. Thus, gender characteristics play an important role in similarly focused research [49]. In this area, it is also appropriate to take into account income, which may be related to the psychological well-being of young-adult students [50], while in several studies, the PSS score increased with lower income of the general population [51,52].

Accordingly, it is clear that stress is a key factor in examining individuals’ mental health and behavioural patterns, and the PSS diagnostic tool can contribute its informational value. Cohen’s perceived stress scale correlated with depressive and physical symptomatology, social anxiety, but also the use of health care [31]. Ruisoto et al. [53] also revealed that the PSS score was positively correlated with several health indicators, including depression, anxiety, and alcohol consumption. Consequently, the increased PSS score was related to the problem of alcohol abuse among university students [48]. 

Similar findings were presented in other studies that examined students’ perceived stress using the 10-item PSS tool, and many confirmed a correlation with depression as measured by the Patient Health Questionnaire (PHQ) tool [54] or Beck’s Depression Inventory [55]. Regarding the research in this study, the findings of Kaya [56] should be highlighted, as these findings show that students with higher levels of depression, as measured by the 9-item version of the PHQ tool, had significantly higher levels of perceived stress, as measured by the 10-item PSS tool. As a result, the author [56] identified perceived stress as the most important determinant of depression in university students. This statement is also consistent with the findings of Zajenkowska et al. [57], who revealed that Korean university students reported higher levels of depression (PHQ 9) due to higher levels of perceived stress (PSS 10) compared to Polish students. Additionally, Hou et al. [58] not only confirmed that the perceived stress score of the 14-item PSS tool was positively related to depression and anxiety, but the authors also found that depression and anxiety were positively related to the problematic use of social networking sites. Thus, depression and anxiety mediated the relationship between perceived stress and behavioural disorder.

From the above-mentioned findings, it can be stated that depression and health-related behavioural disorders play an important role in the study of perceived health. Therefore, depression and alcohol use disorder are theoretically discussed below.

### 2.2. Depression

Depression is a widespread mental disorder across the general population and can lead to serious problems in the lives of individuals [59]. In any case, university students are at a higher risk of depression than the general population, as evidenced by a prevalence rate of this disorder in students ranging from 10% to 85% [6]. This may be related to the unbalanced lifestyle of students, who are characterized by a lack of sleep, a lack of quality food, or a lack of time spent with family [60,61]. At the same time, the most serious concerns of depressed students include academic performance, pressure to succeed, and post-graduation plans [62]. Thus, it can be stated that the most at-risk population group in terms of depressive disorders are students [63], especially female students who have a higher rate of depression than their male counterparts [49]. At the same time, it was confirmed that a prevalence of depression in students increased during their university studies [64].

Due to its brevity and verified psychometric properties, the Patient Health Questionnaire (PHQ) appears to be a very suitable tool for measuring depression [65,66], while Kroenke a Spitzer [67] emphasized the dual purpose of the 9-item version (PHQ 9), first, to diagnose depression and, second, to assess the severity of depression in individuals. This tool has proven successful in many studies focused on screening for depression in a sample of university students [57,68,69,70]. Subsequently, Grant et al. [71] found that the risk of depression (PHQ 9) in students under stress increases. Surprisingly, it was also found that non-medical students reported higher PHQ 9 scores than medical students [72], while based on the above-mentioned findings on stress in medical students, the opposite findings were expected. Lipson et al. [73] also revealed that students in the humanities, arts, and design disciplines are more likely to have mental health problems, such as depression, anxiety, suicidality, or self-injury.

In a sample of Slovak university students, this tool was used by Hajduk et al. [74], who found a mean PHQ 9 score of 8.54 and identified 35.5% of respondents as students with depression. These authors [74] also emphasized that Slovak students are characterized by a higher prevalence of depression than anxiety and, simultaneously, students with higher scores tend to perceive their mental health as less satisfactory. When comparing the mean PHQ 9 scores obtained in international studies, mild depression was found among university students in many countries, such as Malaysia [75], Turkey [56], Germany, China [76], Croatia [77], Japan [78], or Colombia [69]. By using this depression-screening tool, female students acquired higher scores than male students [79]. 

Based on these findings, it can be concluded that students often suffer from mild depression and it is necessary to address this disorder. Garlow et al. [68] also emphasized this need, as there is a strong relationship between depressive symptoms (PHQ 9) and suicidal thoughts among university students.

### 2.3. Alcohol Use Disorders

In addition to the high prevalence of depression, it has also been found that depressive disorders in students may be associated with the use of addictive substances such as alcohol, cigarettes, or cannabis, especially in the case of severe depression [80]. Simultaneously, Sebena et al. [81] examined the associations between perceived stress, depressive symptoms, and alcohol consumption in university students from Germany, Poland, Bulgaria, the UK, and the Slovak Republic, and their findings revealed that perceived stress and depressive symptoms were associated with problem drinking. Thus, alcohol consumption significantly enters into the issue of mental health of university students.

Alcohol consumption is a common unhealthy pattern of behaviour among students, and the reasons for drinking alcohol are, for example, relieving stress or forgetting something bad [82]. In any case, excessive alcohol consumption is a very serious problem in universities, as the consequences of this unhealthy behaviour include missed classes and lower grades, injuries, sexual assaults, overdoses, memory blackouts, changes in brain function, lingering cognitive deficits, and death [83]. University students who suffer from problem drinking with the risk of addiction are also characterized by the use of other addictive substances (tobacco, marijuana, or cocaine), but also by depression, psychological distress, risk behaviour, and low interest in academic activities [84]. 

The relationship between alcohol use disorders and major depression is well-known in this research area [85]. Alcohol dependence can be considered a risk factor for the adverse course of depressive disorder [86], on the other hand, depressed moods can be considered a risk factor for problematic alcohol consumption [87]. This provides an opportunity for a more detailed examination of the issue. With a focus on the main idea of the presented study, there is evidence of a significant correlation between alcohol dependence, as measured by the Alcohol Use Disorders Identification Test (AUDIT), and depression (PHQ 9) in a sample of university students [88]. Ndegwa et al. [89] also found that the AUDIT score correlated with the PHQ 9 score, in other words, an increase in alcohol use was associated with an increase in depression. Based on the above-mentioned findings, it can be emphasized that the AUDIT and PHQ 9 tools are commonly used in studies dealing with students’ mental health and their health-related behaviours [90,91,92]. Additionally, it was found that while female students had a higher prevalence of depression (PHQ 9), male students suffered from harmful drinking (AUDIT) [93]. 

As already mentioned, the Alcohol Use Disorders Identification Test is widely used to assess alcohol consumption, which confirms its reasonable psychometric properties in a sample of university students [94,95,96,97,98]. Its outputs also correlate with several health-related measures, including the Cohen’s perceived stress scale and the Patient Health Questionnaire [99]. 

According to the mean of the total AUDIT score, Ecuadorian and Spanish students had a low risk for alcohol use disorder [99,100]. On the other hand, British and Swedish students reported hazardous drinking [101,102]. In the US, the mean AUDIT score was 5.70, female students obtained a value of 4.86, and their counterparts acquired a value of 7.53, indicating a significant gender difference at the expense of male students [103]. With a focus on problem drinking, students in many European countries are no exception, and evidence again shows that this problem mainly affects male students [87,104]. In terms of socio-economic characteristics, not only gender, but also income plays an important role, while students’ higher incomes can lead to their higher alcohol consumption [104]. Using a univariate analysis, Kumar et al. [105] also confirmed that not only the male gender, but also the level of income is significantly associated with the level of alcohol use measured by the AUDIT diagnostic tool. In response to these findings, alcohol use disorders are also a serious problem that needs to be addressed.

Based on the above-mentioned facts, it can be stated that the issue of mental and behavioural disorders is a much-discussed topic in the professional and scientific community. International research teams and their findings emphasize the need to address these health problems in students, especially with regard to stress, depression, and alcohol consumption. Otherwise, the socio-economic consequences in the future will be borne not only by individuals, but also by society as a whole. This was the greatest motivation to carry out the presented research in the Slovak Republic, where this issue is still insufficiently examined. In addition, the COVID-19 pandemic underlines the importance of addressing mental health issues.

## 3. Materials and Methods

### 3.1. Research Objective

As mentioned in the previous section, mental disorders need to be taken with some seriousness. Mental and behavioural disorders are destructive to the individual who suffers from them, but also to society as a whole. The present is defined by globalization, internationalization, or other pressures stemming from development, which may be reflected in certain negatives that cause stress in economic and social life. In this context, stress is a predictor of serious mental disorders, one of which is depression, and it is not uncommon for depressed people to reach for alcohol. The destructive effects of alcohol on the population and the economy are undeniable; and therefore, research into this issue in different conditions and from different perspectives is very beneficial. Accordingly, the main objective of the presented study was to examine the effects of perceived stress on depression and subsequently to examine the effects of depression on alcohol use disorders. The classification of gender and income characteristics was selected for the study.

This objective was achieved through several analytical processes, and in the first step, the data were statistically described with a primary focus on gender and income differences. In the second step, the main part of the research was carried out, which evaluated the effects of perceived stress on depressive disorder and subsequently the effects of this disorder on alcohol use disorder. The specificity of the research lies in the sample, which was characterized by distance education (study at home using information and communication technologies) during a pandemic associated with the severe acute respiratory syndrome coronavirus 2 (SARS-CoV-2), i.e., the coronavirus disease 2019 (COVID-19).

### 3.2. Research Sample and Data Collection Process

The research sample consisted of Slovak university students, and primary data were collected from an electronic questionnaire distributed during the COVID-19 pandemic using the Google Forms tool. Thus, the analysed population consisted of all Slovak university students. Prior to data collection, the numbers of universities, their approximate size based on the number of students, and fields of study were mapped. The requirements for the selection of the research sample were determined by quota sampling, while in the first step, the ambition was to include most universities in the research. After removing irrelevant data, the sample selection represented 31 of 34 (current) universities, with an average of 48 observations per university. Another requirement was the inclusion of each field of study, while a condition was set for the acquisition of more than 30 observations per study field. Gender characteristics also played a role in this context: Education: *N* = 73 (4.79%), F = 75.34%, M = 24.66%; Humanities and arts: *N* = 67 (4.40%), F = 82.09%, M = 17.91%; Social, economic, and legal sciences: *N* = 608 (39.92%), F = 71.05%, M = 28.95%; Natural sciences: *N* = 62 (4.07%), F = 61.29%, M = 38.71%; Design, technology, production, and communications: *N* = 152 (9.98%), F = 21.71%, M = 78.29%; Agricultural and veterinary sciences: *N* = 47 (3.09%), F = 68.09%, M = 31.91%; Health services: *N* = 164 (10.79%), F = 85.37%, M = 14.63%). The selection of the sample was also conditioned by the degree of study, the year of study, and the form of study, the frequencies of which are given in Table 1.

The data collection was carried out by contacting the representatives of Slovak universities (rectors, vice-rectors, deans, vice-deans) and administrative staff (who are in contact with students) by e-mails requesting the distribution of the questionnaire among students through their university e-mail addresses. Simultaneously, delegates of the student council for higher education were approached with a request to distribute the questionnaire among students (through their university social networks). After the first round of collection, a basic analysis of the data and their frequencies was performed. In the second round, the data collection was focused on specific categories of students, meaning that e-mails requesting the distribution of the questionnaire to students with individual specifications were sent to university staff (in this case also to teachers who had e-mails published on university websites).

A total of 1823 observations were collected from respondents. In the introduction of the questionnaire, it was possible to agree/disagree with participation in the research, which allowed respondents to confirm their informed consent. Respondents who did not agree to participate in the research were not included in this number. The questionnaire also included a control item indicating agreement with the statement that one million has six zeros (a numerical expression was also provided), respondents who did not agree with this statement were excluded from the research. Students with other than Slovak nationality were also excluded, as well as Slovak students who stated that they were primarily studying in another country, for example in the Czech Republic (these were individuals to whom the questionnaire was distributed at random and they probably studied both in the Slovak Republic and in the Czech Republic). The validity of the observations was also assessed on the basis of obviously irrelevant responses to the questionnaire items. A total of 350 respondents were excluded and the research sample consisted of 1523 respondents.

Table 1 shows the characteristics of the respondents in terms of selected identification variables. The Slovak system of universities is organized into three degrees of study, the first degree represents a bachelor’s study (3 years in full-time form), which is followed by a master’s (or engineering) study (2 years in full-time form) as the second degree, and the last third degree represents a doctoral study (3 to 4 years in full-time form). The combination of the first and second degree represents a specific form that is characteristic of fields of study, such as medical fields (5 to 6 years). 

In this research, the most frequent category was the 1st degree (*n* = 1053; 69.1%), which was expected as (i) many disciplines do not have the opportunity to study in the second degree, and (ii) a certain proportion of students do not continue in the second degree. The most frequent form of study was the full-time form (*n* = 1417; 93%) and the less frequent form was the part-time form (*n* = 106; 7%), in which it is necessary to pay a fee and usually study longer. The gender variable represented 63.7% females (*n* = 970) and 36.3% males (*n* = 553). According to the median, the income variable was divided into two categories: Less than 160 EUR (inclusive) and more than 160 EUR. A combination of these gender and income categories was used in the analyses, namely: ≤160 Female (*n* = 521; 34.2%); ≤160 Male (*n* = 247; 16.2%); 161+ Female (*n* = 449; 29.5%); 161+ Male (*n* = 306; 20.1%). Based on the above-mentioned, it can be assumed that the sample is representative for the population in question, despite minor variations.

The design and realization of the study were approved by the ethics committee of the General University Hospital in Prague as individual research (Ref. 915/20 S–IV). All respondents included in this study confirmed informed consent in the questionnaire. The informed consent included a brief description of the research and information on stakeholders. The rights and anonymity of the respondents were also emphasized. The respondents were informed that there were no right and wrong answers, only attitudes, and they were asked to complete the questionnaire responsibly. At the end of the informed consent, there was a question item regarding the reading and understanding of the previous text, in which the respondents were assured that everything is in accordance with the current legal decree, and whether they agreed to participate in the research. An email address was provided in the informed consent. All aspects in this research were conducted with respect to the seventh revision of the World Medical Association–Declaration of Helsinki [106] and the second revision of the Farmington Consensus [107]. 

### 3.3. Research Instruments and Variables

The analytical process included three variables representing the symptoms of selected mental and behavioural disorders, as well as variables of gender specification and income. Three variables associated with mental and behavioural disorders were obtained using diagnostic tools such as the Perceived Stress Scale (PSS), the Patient Health Questionnaire for depression (PHQ 9), and the Alcohol Use Disorders Identification Test (AUDIT). The PSS diagnostic tool was selected on the basis of its extensive use in the professional and scientific community, as mentioned in the theoretical background. The authors of this tool [31,108] presented a 14-item version in their research. This version was later modified to a 10-item scale, and this modified version was used in this research. The PHQ 9 diagnostic tool was developed and validated by Kroenke, Williams, and Lowe [65,66] and, as pointed out in the theoretical background, it can be considered a tool accepted by the professional and scientific community. The AUDIT diagnostic is also an accepted tool for identifying the intensity of alcohol use disorders [109]. 

The items of the PSS tool included the following categories: Never (0), almost never (1), sometimes (2), often (3), very often (4), and the resulting score was the sum of the coding values (low stress (0–13), moderate stress (14–26), high stress (27–40)). The PHQ 9 items were composed of the following categories: Not at all (0), several days (1), more than half the days (2), nearly every day (3), and the resulting score was the sum of the coding values (none (0–4), mild (5–9), moderate (10–14), moderately severe (15–19), severe (20–27) depression). The AUDIT items were numerically scored from 0 to 4, and the resulting score was the sum of the individual items, which was transformed into zones representing the intensity of alcohol use disorders (ZONE I (low risk): Alcohol education (0–7); ZONE II (hazardous use): Simple advice (8–15); ZONE III (harmful use): Simple advice plus brief counselling and continued monitoring (16–19); ZONE IV (dependent use): Referral to specialist for diagnostic evaluation and treatment (20–40)). The numeric indices for abbreviations determine the number of items that each diagnostic tool contains.

### 3.4. Statistical Analysis

The methods of descriptive and inferential statistics were primarily used for analytical processing. In the first step, a descriptive analysis was used, in which several statistical characteristics (mean (Mean), confidence interval (95% CI), median (Median), standard deviation (Std. Deviation)) were used to describe the variables determining mental and behavioural disorders (PSS, PHQ 9, AUDIT) without classification, but also in the classification of gender and income categories of students (females, males, ≤160 EUR, 161+ EUR). Subsequently, an analysis of differences was performed using non-parametric tests (Wilcoxon rank sum test with continuity correction; Kruskal–Wallis rank sum test). These tests were used to identify differences, and thus to confirm the relevance of including gender-income characteristics in subsequent analyses. In terms of practical implications, the purpose of these tests was to identify the group with the highest score in a particular mental or behavioural health variable, and thus in which group the intervention effort is most needed. A correspondence analysis was also used in the descriptive part of the analytical processing, the results of which complete the information on the intensity of disorders (in intervals) in connection with gender and income characteristics (≤160 Female; ≤160 Male; 161+ Female; 161+ Male). The gender-income categories were also included in the part of the analytical processing, in which a regression analysis (simple quantile regression analysis) and a correlation analysis (Spearman’s ρ) were applied. The purpose of these analyses was to point out the relations between the PSS score and the PHQ 9 score, as well as the relations between the PHQ 9 score and the AUDIT score in terms of the classification of the analysed gender and income characteristics. Analytical calculations were performed using the programming language R v 4.0.2 (RStudio, Inc., Boston, MA, USA) nickname: Taking off Again.

## 4. Results

This section is devoted to the interpretation and description of the findings resulting from the applied analytical processes. This section can be divided into two main parts, where the first part consists of descriptive analyses in order to present the variables in more detail. The second part of the analyses focuses on the evaluation of the relationships between selected variables. These analytical processes help to create a clear picture of the examined issue, to understand the problem, and to draw conclusions.

### 4.1. Descriptive Part

The analytical processes included three main variables, namely (i) the PSS score, (ii) the PHQ 9 score, and (iii) the AUDIT score, which are described in Table 2 (based on selected statistical characteristics). The characteristics in this table provide information on the output of the analysed variables as a whole (without classification), as well as in the classification of gender categories (Female and Male) and in the classification of income categories (≤160 and 161+).

The PSS score theoretically assumes a maximum value of 40, while a value of up to 13 represents a low level of stress. With a focus on the average values in this analysed case, it is possible to see a relatively acceptable rate. In terms of gender characteristics, a significantly higher value of central tendencies was found in female students (95% CI: Female = 6.77–7.19; Male = 3.88–4.34), and in the case of income categories, a higher value was identified in the category of students with lower income (95% CI: ≤160 = 5.88–6.38; 161+ = 5.51–6.01). 

The PHQ 9 score can theoretically reach a maximum value of 27, and a value higher than 4 indicates an increased level of mental depressive disorder. When focusing on gender characteristics, a slightly higher value of central tendencies was measured in females (95% CI: Female = 5.42–6.09; Male = 5.21–6.16) and in terms of income, a higher value was identified in the category of students with lower income (95% CI: ≤160 = 5.58–6.37; 161+ = 5.11–5.87). 

From a theoretical point of view, the AUDIT score can reach a maximum value of 40, while a value of up to 7 defines ZONE 1, which represents a low risk. With a focus on gender characteristics, a significantly higher value of central tendencies was found in male students (95% CI: Female = 4.87–5.42; Male = 7.05–8.02) and in terms of income categories, a higher value can be observed in the category of students with higher income (95% CI: ≤160 = 5.41–6.11; 161+ = 5.88–6.63).

In order to provide more detailed information on the PSS and PHQ 9 outputs and their link to the AUDIT score, Table 3 shows the arithmetic mean and median of the AUDIT score in each combination of the PSS and PHQ 9 intervals. Based on the results, it can be observed that lower values of the AUDIT score were attributed to the intervals with a lower level of mental disorders (PSS, PHQ 9) and vice versa (Mean: PSS–Low and PHQ 9–None = 5.54; PSS–High and PHQ 9–Severe = 9.35). At the same time, there were some deviations from this trend, for example in the combination of PSS–Moderate and PHQ 9–None (Mean = 5.20; Median = 4.00).

Table 4 provides the results of the analysis of the differences in the PSS, PHQ 9, and AUDIT scores between gender and income characteristics. As can be seen, significant differences were found in most of the analysed cases. Only in two cases (PHQ 9–Gender; AUDIT–Income), it is not possible to confirm the difference at a significance level lower than 0.05.

**Figure 1:**
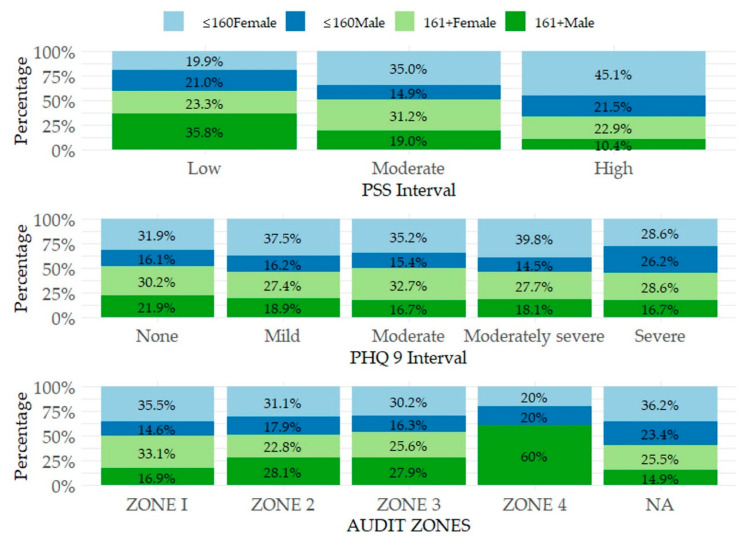
Distribution of PSS, PHQ 9, and AUDIT intervals in terms of gender-income categories.

Figure 1 shows the distribution of adjusted outputs (intervals) in the variables PSS, PHQ 9, and AUDIT. When focusing on the PSS variable, which was divided into three intervals, it is possible to observe a small part of the research sample in the low and high intervals (Low: *n* = 176, 11.56%; High: *n* = 144; 9.46%). In contrast, the moderate interval of PSS covered most of the research sample (Moderate: *n* = 1203, 78.99%). It is also possible to focus on the distribution of these stress intervals in terms of gender-income characteristics. In the low interval, the dominant category was male students with higher incomes (161+ Male: 35.8%) and the least frequent category was female students with lower incomes (≤160 Female: 19.9%). In the moderate interval, female students dominated, and in the case of the high interval, the most frequented category were female students with lower incomes (≤160 Female: 45.1%). 

The PHQ 9 score was divided into 5 intervals indicating the level of depressive disorder. Based on the data in this research, respondents included in the none interval (without depressive disorder) represented 51.94% (*n* = 791) of the research sample, respondents included in the mild interval represented 29.22% (*n* = 445) of the sample, respondents included in the moderate interval represented 10.64% (*n* = 162) of the sample, respondents included in the moderately severe interval represented 5.45% (*n* = 83) of the sample, and finally 2.76% (*n* = 42) of the research sample were respondents included in the severe interval. In terms of gender-income characteristics, the distribution was similar in several intervals, while the dominance of female students was evident. In the severe interval of PHQ 9, a certain level of proportionality can be observed for the female categories (≤160 Female: 28.6%; 160+ Female: 28.6%) and for the male category with lower incomes (≤160 Male: 26.2%).

In general, the AUDIT score can be assigned to one of four zones reflecting the intensity of alcohol use disorders. In this study, the ZONE 1 represented 72.87% (*n* = 1007) of the research sample, the ZONE 2 included 21.85% (*n* = 302) of the sample, the ZONE 3 included 3.11% (*n* = 43) of respondents, the ZONE 4 included 2.17% (*n* = 30) of respondents, and 9.30% (*n* = 141) of the research sample were students who stated that they had not drunk alcohol at all in the last year. By focusing on the distribution of intervals in terms of gender-income characteristics, female students dominated in the ZONE 1. Both the ZONE 2 and the ZONE 3 were dominated by female students with lower incomes as well as male students with higher incomes. A significant dominance of male students with higher incomes can be observed in the ZONE 4 interval (161+ Male: 60%).

The following part of the analytical process is focused on assessing the significance of selected relations. A correspondence analysis was used, which enriched the findings on the differences identified in the previous analysis.

Table 5 shows the assumptions for the application of correspondence analysis. In this analysis, the relations between gender-income categories and disorder categories (PSS, PHQ 9, and AUDIT intervals) were assessed. With a focus on the results, the most important information is provided by the *p*-value (Sig.) of the χ^2^ test, which can be observed in the last column of the table. Obviously, the significance was not confirmed only in the relation between gender-income and PHQ 9. On the other hand, the significance was clearly confirmed in the other two relations. Another important information of Table 5 is shown in the column Variance%. In the relation between gender-income and PSS, the first dimension explains 81.12% of the variability, and in the case of the relation between gender-income and AUDIT, the first dimension is even more dominant, i.e., 99.25%.

**Figure 2:**
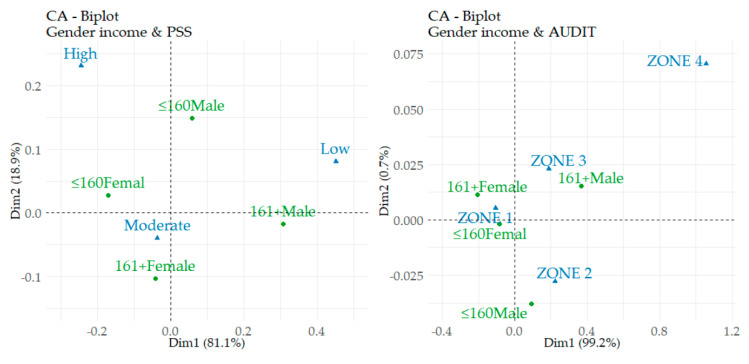
Correspondence analysis map.

Figure 2 shows the links between the categories of analysed variables (gender-income categories, interval disorder categories). In the case of gender-income and PSS, the moderate and high intervals of perceived stress are close to the categories of female students and the category of male students with lower incomes. In contrast, the low stress interval is closest to male students with higher incomes. In the case of gender-income and AUDIT, a certain indentation of the ZONE 4 is evident. When interpreting the other zones, a close link can be identified between the category of male students with higher incomes and the ZONE 3. Conversely, the category of male students with lower incomes is close to the ZONE 2. Finally, both categories of female students are concentrated around the ZONE 1.

### 4.2. Relational Part

This part of the analytical processes is focused on evaluating the significance of selected relations. A regression analysis was used, the results of which can be considered very valuable in terms of achieving the main objective of this study. The conclusion of this part is devoted to the application of correlation analysis, which completes the idea of the significance of the assumed relationships.

Table 6 provides the results of a quantile regression analysis that was used to assess the effects of perceived stress (PSS) on depression (PHQ 9) and the effects of depression (PHQ 9) on alcohol use disorders (AUDIT) in terms of gender and income characteristics. In the first step, it is appropriate to focus on the effects of the PSS score on the PHQ 9 score. Based on the results, the significance was confirmed in all of the analysed relations. The independent variable (PSS) showed values with a positive coefficient. Accordingly, an increase in the PHQ score can be expected as the PSS score increases.

Focusing on the second part of Table 6, the significant effects of the PHQ 9 score on the AUDIT score were found in most of the analysed cases. At the same time, the significant effects could be observed in all gender-income categories with the highest AUDIT score (λ = 0.75). The trajectory of the PHQ 9 coefficients is also positive in all cases, indicating the fact that if the PHQ 9 score increases, the AUDIT score can also be expected to increase. 

Appendix A shows the analysed relations, while the visualized effects can be interpreted in such a way that the steeper the regression line, the stronger the effect. In general, it can be concluded that the effects of perceived stress (PSS) on depression (PHQ 9) were stronger than the effects of depression (PHQ 9) on alcohol use disorders (AUDIT). 

A clear picture of the examined issue is completed in Table 7, which provides the results of the analysis of relationships (correlation analysis). A significant relationship was found in all of the analysed cases, while closer relationships can be observed for PSS and PHQ 9 than for PHQ 9 and AUDIT. With a focus on PSS and PHQ 9, the vast majority of cases can be considered substantial to very strong relationships. Only one case did not correspond to this, in which a moderate to substantial relationship was identified (161+ Female: ρ = 0.484). With a focus on PHQ 9 and AUDIT, it is possible to observe a low to moderate rate of relationships. Positive coefficients were identified in all of the analysed cases, indicating that an increase in depression (PHQ 9) may be associated with an increase in stress (PSS). At the same time, an increase in alcohol use disorders (AUDIT) may be associated with an increase in depression (PHQ 9). However, the presented trajectory also applies the opposite, which would be a better case for Slovak university students.

## 5. Discussion

University students are characterized by vulnerabilities related to their mental health and unhealthy patterns of behaviour [1,7,8]. The threat is burnout [27], stress [4,5], depression [6,63], but also behavioural disorders [83,84]. For these reasons, it is necessary to pay attention to university students in an effort to help them overcome their difficulties. The results of our analyses have revealed interesting findings, which are discussed in the following part with many international studies.

### 5.1. Prevalence and Levels of Stress, Depression, and Alcohol Use Disorders of Slovak Students in an International Comparison

In this study, it was generally found that Slovak university students reported acceptable PSS, PHQ 9, and AUDIT scores. When comparing the obtained PSS score, it is possible to point out the fact that Slovak students reported a lower level of perceived stress than students in other countries. Specifically, Slovak students acquired a mean PSS score of 5.95, which is obviously less than a mean value of 18.43 for Korean students [30], 19.79 for students from the UK [47], or 32.2 for Italian students [46]. In terms of gender specification, a higher mean PSS score was found in female students, which is consistent with the findings revealed in the US study [45].

With a focus on the mean value of the total PHQ 9 score (mean = 5.73), it is possible to observe a mild level of depression in Slovak university students. Hajduk et al. [74] also found that Slovak students suffer from mild depression (mean = 8.54). Thus, Slovak students are comparable to students from Croatia (median = 6) [77], Germany (mean = 6.77), China (mean = 6.99) [76], Turkey (mean = 7.39) [56], or Malaysia (mean = 8.10) [75], who reported mild depression. Again, female students acquired a slightly higher score than male students, but a significant difference was not confirmed, which is consistent with the results of the Japanese study [78]. On the other hand, greater gender differences in scores were found in a study conducted by Miranda and Scoppetta [69].

The mean value of the total AUDIT score showed a low intensity of alcohol use disorders in Slovak students (mean = 6.01), which corresponds to the results in the US study (mean = 5.70) [103]. Ecuadorian and Spanish students also reported a low risk of alcohol use disorder [99,100]. On the contrary, our findings are not consistent with the findings of the UK study in which students reported hazardous use (ZONE II on the AUDIT scale) (mean = 9.9) [101]. Slovak female students obtained a mean score of 5.15 and male students obtained a mean of 7.54, which confirms the dominance of males. In other studies, male students also obtained higher AUDIT scores compared to their counterparts [99,100,102].

Based on the output of the analysis of differences, it was possible to confirm significant differences between the gender-income categories in most of the analysed cases. Specifically, significant differences in the PSS score were observed in all cases. In terms of gender specification, female students suffer from stress more than male students, which is in line with the findings of many studies [30,44,46,48]. Drachev et al. [41] also stated that in addition to the socio-economic status of students, gender characteristics also play an important role in similar research involving the PSS diagnostic tool. This study also confirmed that students with lower incomes obtain higher PSS scores, which is consistent with the findings of other authors such as Cohen and Janicki-Deverts [51] or Klein et al. [52]. Accordingly, it is possible to agree with the authors Zhang and Henderson [110], who argued that finance is a significant stress factor. Regarding the PHQ 9 score, the differences were not as obvious as in the previous case. A significant difference (at the level of 0.01) was found only in the income characteristics, while a higher PHQ 9 score was observed in students with lower income. In the case of the AUDIT score, it was possible to speak of a significant difference in gender characteristics with the dominance of male students.

Based on the interval-adjusted outcomes, this study also revealed that 78.99% of Slovak university students suffer from moderate stress, and high stress was identified in 9.46% of students. Similar results were measured in Russian students, who reported low, moderate, and high stress in a prevalence of 26.0%, 69.1%, and 4.9% [41]. On the other hand, Kupcewicz et al. [43] revealed that 41.3% and 45.4% of Slovak nursing students suffered from moderate and high stress, and simultaneously, 49.8% of Polish nursing students and 43.8% of Spanish nursing students reported high levels of the PSS stress score. Additionally, Orosova et al. [39] identified 24.7% of Slovak students with higher levels of stress using the 4-item Cohen’s PSS tool.

Focusing on the PHQ 9 score, Hajduk et al. [74] identified 35.5% of Slovak students with depression. Our findings showed that 10.64%, 5.45%, and 2.76% of Slovak students suffered from moderate, moderately severe, and severe depression, which can be compared with Chinese students who reported moderate to severe depressive symptoms with a prevalence of approximately 11% [111].

Finally, with a focus on the AUDIT score, 21.85%, 3.11%, and 2.17% of students were at-risk alcohol users included in ZONE 2 (hazardous use), ZONE 3 (harmful use), and ZONE 4 (dependent use). In comparison, 8% of Australian university students used alcohol at harmful levels (ZONE 3) and 33% used alcohol at hazardous levels (ZONE 2) [93]. Slovak students also obtained more positive alcohol-related outcomes compared to students from the UK, who were characterized by problem drinking in a prevalence of up to 60.6% (ZONE 2 = 40.1%, ZONE 3 = 10.9%, ZONE 4 = 9.6%) [101].

The results of the correspondence analysis of the relations between the categories of analysed variables (gender-income categories, interval disorder categories) point to the fact that female students in both income categories and male students with lower incomes reported higher levels of perceived stress than male students with higher incomes. By comparing the relations between the gender-income characteristics and the AUDIT score, it was possible to positively assess the fact that a very weak link was found in ZONE 4. In the case of ZONE 3, a close link was found with male students with higher incomes. Both income categories of female students were concentrated around ZONE 1. These findings confirmed the fact outlined above that gender and income characteristics play an important role in the study of mental and behavioural disorders in university students.

### 5.2. Effects within the Analysed Mental and Behavioural Disorders

The output of the regression analysis provided information on the significance of the effects of perceived stress (PSS) on depression (PHQ 9) and the effects of depression (PHQ 9) on alcohol use disorders (AUDIT) in terms of gender and income characteristics. Based on this output, it can be concluded that significant effects were found in most of the analysed cases. The predictors have acquired positive values, which means that if the level of perceived stress (PSS) increases, an increase in the intensity of depressive disorders (PHQ 9) can be expected. Simultaneously, an increase in the intensity of alcohol use disorders (AUDIT) can be expected with an increase in depressive disorders (PHQ 9). Focusing on the strength of the effects, it can be stated that the effect of the PSS score on the PHQ 9 score was stronger than the effect of the PHQ 9 score on the AUDIT score. The presented facts were also confirmed by the results of correlation analysis, on the basis of which it was possible to observe significant positive correlation rates, which indicate a significant to very strong association for PSS and PHQ 9 and a low to moderate association for PHQ 9 and AUDIT. All of these findings are consistent with the findings of many international studies focusing on the mental and behavioural disorders of university students and the identification of their perceived stress, depression, and alcohol use disorders using the PSS, PHQ 9, and AUDIT tools. Specifically, Kaya [56] and Zajenkowska et al. [57] confirmed that the PSS score of university students is a significant predictor of depression measured by the PHQ 9 diagnostic tool. Our findings on the relations between depressive disorder and alcohol use disorder can also be compared with other studies, in which a significant correlation between the AUDIT score and the PHQ 9 score was confirmed [88,89]. In response to these findings, mental health was a major predictor of alcohol use disorders.

According to our findings, it is possible to contribute to the general conclusions of many authors that perceived stress is positively related to depression [54,55,58], and at the same time, depression is associated with the use of addictive substances such as alcohol [80,87]. In this sense, it can be concluded that depression mediated the relation between perceived stress and alcohol use disorders in university students, while Hou et al. [58] found similar evidence in relation to students’ perceived stress, depression, and problematic use of social networking sites. Moreover, this finding of our study is consistent with the fact that students’ perceived stress is associated with their problematic drinking [48,81].

### 5.3. Practical Implications and Interventions

The findings of our study represent a valuable platform for the development of various intervention and prevention programs aimed at university students as an important group of the population. Mental health and unhealthy patterns of behaviour of university students are a frequently discussed topic, underlining the need to pay special attention to this vulnerable population, especially during the COVID-19 pandemic. This period associated with isolation may adversely affect them in terms of increased stress, anxiety, or depressive thoughts [23]. As already mentioned, these difficulties can affect students’ health, well-being, and their patterns of health-related behaviour [24,25,26,28,29,68,83,84]. Last but not least, it is possible to consider mental and behavioural disorders in terms of their socio-economic consequences, which affect individuals, their families, but also society as a whole. The economic burden of these disorders is considerable, which is reflected in increased direct and indirect costs, such as lost productivity and health-related costs [10,11,12,13,14,15,17,18]. For these reasons, stress, depression, and alcohol consumption should not be overlooked. The results of our study highlight the importance of developing intervention and prevention strategies to address the problems of university students in terms of their mental health and health-related behaviour. In this context, it is necessary to take into account the presented connections between individual gender and income categories and a specific disorder. Female students are more prone to stress, male students dominate in alcohol disorders

During the COVID-19 pandemic, the population struggles not only with the coronavirus disease, but also with various aspects that cause stress and depression. This fact underlines the importance of this issue and the processes in this situation. The severe acute respiratory syndrome coronavirus 2 (SARS-CoV-2) is a new disease that is constantly being studied, and society is gradually informed about its consequences and new findings. Therefore, it is important to find a way to eliminate the symptoms of stress and depression that result from this disease and its circumstances. Public advice, guidelines, and recommendations can be very helpful. On this basis, universities, including Slovak ones, should create various programs and inform about the possibilities of support and assistance to students who need help or would like to learn to manage various symptoms of stress and depression in a situation associated with the COVID-19 pandemic.

University counselling centres play a key role in this difficult situation and have great potential to provide professional assistance to students in improving their mental health [112]. Communication strategies and confidence-building have an irreplaceable place not only in universities, but also in public institutions, hospitals, or community centres, which should not forget the mental health of the population and provide guidance on how to manage and overcome stress or perceived mental problems. In this sense, it is appropriate to establish effective programs and interventions to help vulnerable groups [113].

It is also necessary to support students’ efforts to seek help from mental health professionals and to ensure access to mental health care [114]. Psychologists and psychiatrists are available for this purpose, as well as e-counselling and helplines operating 24 h a day. In addition to psychotherapy and treatment, public debates and education on the importance of mental health are very important. Internet-based interventions to reduce stress and mental health problems are also a great opportunity for students who do not seek professional help themselves [36]. Promising interventions may include useful information on websites and social networks, online lectures with experts, online campaigns and challenges aimed at good mental health, or promotional videos from the perspective of young people.

Last but not least, mental health services in the Slovak Republic are underfunded and have limitations resulting from totalitarian history [115,116]. Therefore, there is a great opportunity to improve mental health policy [117]. Political attention should be paid not only to the physical, but also to the mental health of the population, which has its socio-economic value. In this sense, strategies and funding should be strengthened, with Non-Governmental Organizations (NGOs) playing an important role [118]. The main challenges for policy makers are to remove various barriers and to develop effective intervention strategies. The first policy effort should therefore focus on implementing mental health into general health policy, improving public awareness of mental health and reallocating resources towards high-priority mental health needs and vulnerable groups [119].

This study emphasizes the importance of prevention, the availability of information on coping with stress and depression, or information on appropriate counselling centres. Promoting an active lifestyle will continue to play a role in this issue. At the same time, the results of this study suggest that all the above-mentioned interventions may be helpful in reducing alcohol use disorders in university students. This is considered to be very beneficial, as alcohol consumption is also a major problem that society should address. This burden is also evidenced by economic costs attributable to alcohol use and alcohol use disorders [19,20,21,22]. It is therefore important to reduce alcohol consumption in the population in the interests of economic prosperity and well-being. With a focus on university students, there are many ways to improve their health-related patterns of behaviour, in which education for healthy lifestyle, prevention, and assistance to students with problem alcohol use are irreplaceable. Policy makers can also use other effective tools to achieve the health potential of university students, such as high prices and taxes on alcoholic beverages or banned alcohol promotion.

In conclusion, it should be noted that if an increase in mental disorders can lead to an increase in alcohol use disorders, it should be borne in mind that a decrease in mental disorders can lead to a decrease in alcohol use disorders. Society as a whole should address these difficulties, and alcohol-related programs must take into account the mental health of university students.

## 6. Conclusions

Mental and behavioural disorders have significant negative social and economic effects, the importance of which is not sufficiently emphasized in the research areas. On the other hand, stress and depression are becoming more common in the population, and various critical situations play an important role. At the same time, alcohol-related pathological behaviour leads to destructive changes not only for individuals, but also for society. For these reasons, research efforts in this issue appear to be both beneficial and necessary, especially at a time when new determinants of mental health difficulties are emerging, with the global epidemiological crisis of COVID-19 being no exception. This situation requires the strengthening of mental health in terms of its value.

On this basis, the main objective of the study was to examine the effects of perceived stress on depression and subsequently to examine the effects of depression on alcohol use disorders. Within this objective, the classification of gender and income characteristics was selected for the research. This objective was achieved by several analytical processes divided into two parts. In the first part, data were statistically described with the primary focus on gender and income differences. Subsequently, in the second part, the relations between perceived stress, depression, and alcohol use disorder were assessed. The study was performed under specific conditions caused by the COVID-19 pandemic.

The results of the analyses emphasize the undeniable importance of research in the field of mental health and risky behaviour of university students. It is possible to speak of a significant frequency of perceived stress, depression, and alcohol use disorders, as well as significant differences between gender and income categories. Within the analysed variables, positive effects and relationships were also confirmed.

In terms of the interventions, it would be procedurally very difficult to focus on the population as a whole, but systemic recommendations were also discussed. Vulnerable groups need the most attention, and it is important to create an effective system in order to eliminate the psychological burden in their lives. Such a group are also university students who represent the future driving force of the economy, and therefore their psychological well-being and patterns of health-related behaviour should be taken into account today. Underestimating this issue may have many negative effects not only on individuals and their future development, but also on society. Prevention and education, the availability of information on mental and behavioural disorders, and the promotion of a healthy lifestyle are essential for success.

### 6.1. Limitations of the Study

The findings of our study should be considered in light of its limitations. A possible limitation is the fact that in the case of non-random sampling, there is a certain risk of insufficient representativeness of the research sample. However, our selection of the research sample (quota sampling) was the most appropriate alternative under the given conditions of the COVID-19 pandemic. Another limitation is the accuracy of self-report data. We also considered the fact that the distance education could be different and with different demands on the student across fields of study in the Slovak Republic. For this reason, the degree of stressful situations and psychological difficulties associated with this form of education could be different. However, these facts would be very difficult to quantify due to the subjectivity in the perception of each individual, as well as due to the significant heterogeneity of the fields of study represented in our research sample. Endogeneity can also be a potential limitation, as some international studies have examined several aspects of mental and behavioural disorders from the opposite perspective. The interpretation of relations in the causal dimension needs to be considered with some caution. However, these limitations cannot be considered as an element that could have a significant effect on the results of our study.

## 6.2. Future Research

Future research should focus on examining the effects of mental and behavioural disorders on selected determinants in homogeneous fields of study in order to compare them within a defined research dimension. It is expected that it will be possible to identify new determinants that affect differences in mental and behavioural disorders in university students. The purpose will be to form standardizable parameters to ensure an optimal system of distance education at a time of various epidemiological crises in the future. Additionally, our future research ambition is to expand the research sample to include students from other countries and to focus on the problems of addictive behaviour in more detail. Interesting results are expected, as the analyses will also include other country-specific variables, i.e., culture or historical development.

## Figures and Tables

**Table 1:** Frequency analysis of the research sample.

Variable	Frequency	Percent
Degree of study:		
1st degree	1053	69.1
2nd degree	379	24.9
Combined 1st and 2nd	35	2.3
3rd degree	56	3.7
Year of study:		
1st	555	36.4
2nd	475	31.2
3rd	265	17.4
4th	94	6.2
5th	124	8.1
6th	10	0.7
Form of study:		
Full-time	1417	93.0
Part-time	106	7.0

**Table 2:** Descriptive analysis of behavioural and mental disorders.

Descriptive Characteristics		PSS	PHQ 9	AUDIT
All	Mean	5.95	5.73	6.01
	95% CI	5.77–6.12	5.45–6.01	5.76–6.27
	Median	6	4	5
	Std. Deviation	3.33	5.22	4.83
Female	Mean	**6.98**	5.76	5.15
	95% CI	**6.77–7.19**	5.42–6.09	4.87–5.42
	Median	7	4	4
	Std. Deviation	3.24	5.12	4.15
Male	Mean	4.11	5.68	**7.54**
	95% CI	3.88–4.34	5.21–6.16	**7.05–8.02**
	Median	4	4	6
	Std. Deviation	2.62	5.39	5.53
≤160	Mean	6.13	**5.98**	5.76
	95% CI	5.88–6.38	**5.58–6.37**	5.41–6.11
	Median	6	**5**	4.5
	Std. Deviation	3.29	5.28	4.61
161+	Mean	5.76	5.49	6.26
	95% CI	5.51–6.01	5.11–5.87	5.88–6.63
	Median	5	4	5
	Std. Deviation	3.36	5.14	5.03

Note: The highest values of central tendencies in a particular health disorder are highlighted in bold.

**Table 3:** AUDIT (Alcohol Use Disorders Identification Test) score in a combination of Perceived Stress Scale (PSS) and Patient Health Questionnaire (PHQ 9) intervals.

AUDIT			PHQ 9				
			None	Mild	Moderate	Moderately Severe	Severe
**PSS**	Low	M	5.54	6.06	9.00	-	-
		Med	5.00	6.00	9.50	-	-
	Moderate	M	5.20	6.23	6.54	9.30	11.67
		Med	4.00	5.50	5.00	8.00	6.00
	High	M	7.00	6.71	6.88	7.63	9.35
		Med	6.50	5.00	5.00	6.00	6.50

Note: M—Mean; Med—Median.

**Table 4:** Gender and income differences in PSS, PHQ 9, and AUDIT.

Between Variable:	Statistic	PSS (*p*-Value)	PHQ 9 (*p*-Value)	AUDIT (*p*-Value)
Gender	W	212,825.0 (<0.001)	**255,215.5 (0.115** **)**	157,865.5 (<0.001)
Income	W	250,860.5 (<0.001)	265,993.5 (0.007)	**225,313.5 (0.077)**
Gender-Income	Χ^2^	62.01 (<0.001)	9.00 (0.029)	85.58 (<0.001)

Note: W—Wilcoxon rank sum test with continuity correction; χ^2^—Kruskal–Wallis rank sum test; *p*-values higher than 0.05 are highlighted in bold.

**Table 5:** Correspondence analysis output.

Relation	Dimension	Eigenvalue	Variance%	χ^2^ (Sig)
Gender-income & PSS	Dim.1	0.0302	81.12	56.78 (<0.001)
	Dim.2	0.0070	18.88	
Gender-income & PHQ 9	Dim.1	0.0041	55.62	11.10 (0.5205)
	Dim.2	0.0022	29.89	
	Dim.3	0.0011	14.49	
Gender-income & AUDIT	Dim.1	4.43 × 10^−2^	99.25	61.75 (<0.001)
	Dim.2	3.13 × 10^−4^	0.70	
	Dim.3	2.32 × 10^−5^	0.05	

**Table 6:** Quantile regression analysis output.

λ	Coef.	≤160 Female	≤160 Male	161+ Female	161+ Male
**PSS -> PHQ 9**					
0.25	Intercept (sig.)	−4.77 (<0.001)	−4.83 (<0.001)	−4.00 (<0.001)	−2.86 (0.009)
	Predictor–PSS (Sig)	0.38 (<0.001)	0.42 (<0.001)	0.33 (<0.001)	0.29 (<0.001)
0.5	Intercept (sig.)	−5.00 (<0.001)	−4.91 (<0.001)	−4.09 (0.002)	−4.00 (<0.001)
	Predictor–PSS (Sig)	0.50 (<0.001)	0.55 (<0.001)	0.45 (<0.001)	0.50 (<0.001)
0.75	Intercept (sig.)	−5.33 (<0.001)	−6.14 (<0.001)	−6.50 (<0.001)	−3.69 (<0.001)
	Predictor–PSS (Sig)	0.67 (<0.001)	0.76 (<0.001)	0.75 (<0.001)	0.62 (<0.001)
**PHQ 9 -> AUDIT**					
0.25	Intercept (sig.)	2.00 (<0.001)	2.91 (<0.001)	1.50(<0.001)	3.88 (<0.001)
	Predictor–PHQ 9 (Sig)	**0.00 (1.000)**	**0.05 (0.574)**	0.13 (0.004)	**0.06 (0.444)**
0.5	Intercept (sig.)	3.00 (<0.001)	4.88 (<0.001)	3.00 (<0.001)	5.86 (<0.001)
	Predictor–PHQ 9 (Sig)	0.20 (0.002)	**0.12 (0.234)**	0.18 (0.002)	**0.14 (0.201)**
0.75	Intercept (sig.)	5.67 (<0.001)	7.00 (<0.001)	4.60 (<0.001)	8.50 (<0.001)
	Predictor–PHQ 9 (Sig)	0.33 (<0.001)	0.25 (0.021)	0.40 (<0.001)	0.50 (<0.001)

Note: *p*-values higher than 0.05 are highlighted in bold.

**Table 7:** Spearman correlation (ρ) output.

Gender Income	Coefficient	PSS & PHQ 9	PHQ 9 & AUDIT
All	Coef (sig)	0.557 (<0.001)	0.186 (<0.001)
≤160 Female	Coef (sig)	0.573 (<0.001)	0.198 (<0.001)
≤160 Male	Coef (sig)	0.636 (<0.001)	0.141 (0.039)
161+ Female	Coef (sig)	0.484 (<0.001)	0.281 (<0.001)
161+ Male	Coef (sig)	0.569 (<0.001)	0.145 (0.014)

**Figure A1:**
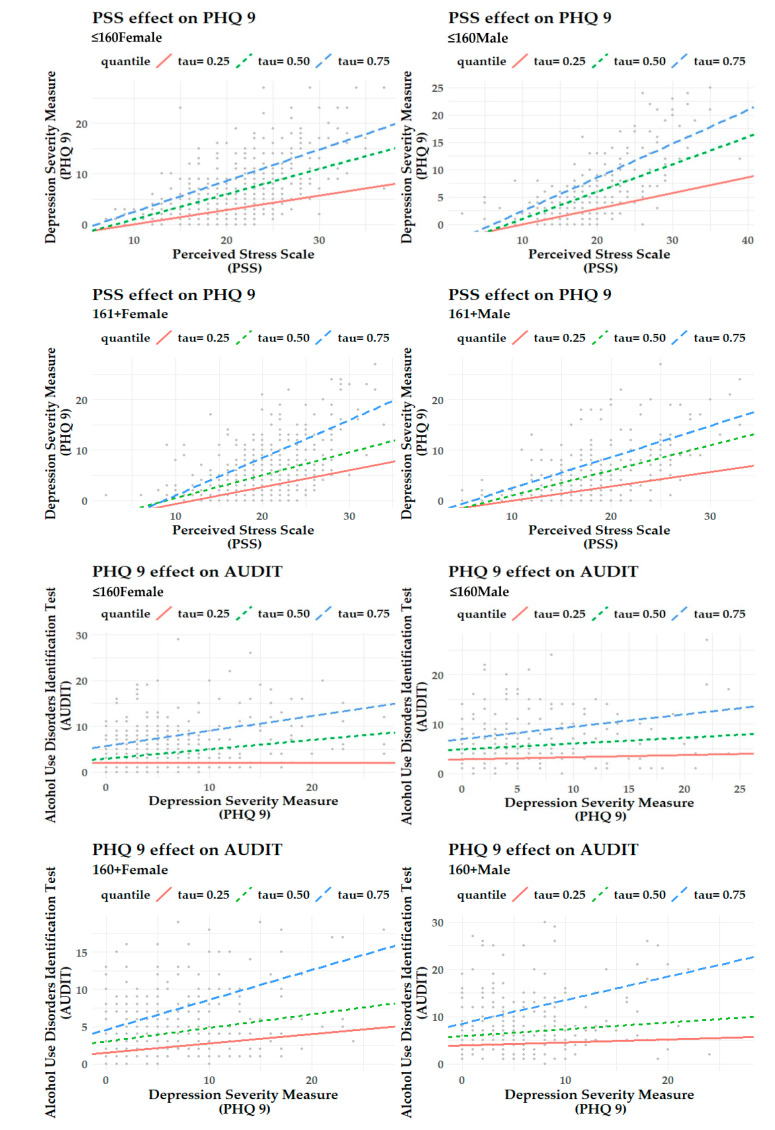
Visualization of effects.
